# The Trace Theorem, the Luzin *N*- and Morse–Sard Properties for the Sharp Case of Sobolev–Lorentz Mappings

**DOI:** 10.1007/s12220-017-9936-7

**Published:** 2017-10-14

**Authors:** Mikhail V. Korobkov, Jan Kristensen

**Affiliations:** 1 Sobolev Institute of Mathematics, Acad. Koptyuga pr., 4, Novosibirsk, Russia; 2 School of Mathematical Sciences, Fudan University, Shanghai, 200433 China; 3 Mathematical Institute, University of Oxford, Andrew Wiles Building, Oxford, OX2 6GG UK

**Keywords:** Sobolev–Lorentz space, Luzin *N*-property, Morse–Sard theorem, Trace theorem, Riesz potentials, Approximation, 58C25, 26B10, 46E30

## Abstract

We prove Luzin *N*- and Morse–Sard properties for mappings $v:{\mathbb {R}}^n \rightarrow {\mathbb {R}}^d$ of the Sobolev–Lorentz class $\mathrm {W}^{k}_{p,1}$, $p=\frac{n}{k}$ (this is the sharp case that guaranties the continuity of mappings). Our main tool is a new trace theorem for Riesz potentials of Lorentz functions for the limiting case $q=p$. Using these results, we find also some very natural approximation and differentiability properties for functions in $\mathrm {W}^{k}_{p,1}$ with exceptional set of small Hausdorff content.

## Introduction

In this paper, we continue the study of the Luzin *N*- and Morse–Sard properties for the Sobolev mappings under minimal integrability assumptions initiated in our previous papers [11, 12, 24], see also [22]. Of course, it is in this context very natural to restrict attention to continuous mappings, and so require from the considered function spaces that the inclusion $v\in \mathrm {W}^k_p({\mathbb {R}}^n,{\mathbb {R}}^d)$ should guarantee at least the continuity of *v*. For values $k\in \{1,\dots ,n-1\}$ it is well known that $v\in \mathrm {W}^k_p({\mathbb {R}}^n,{\mathbb {R}}^d)$ is continuous for $p>\frac{n}{k}$ and could be discontinuous for $p\le \frac{n}{k}$. So **the borderline case** is $p={p_\circ }=\frac{n}{k}$. It is well known (see for instance [22]) that $v\in \mathrm {W}^k_{p_\circ }({\mathbb {R}}^n,{\mathbb {R}}^d)$ is continuous if the derivatives of *k*-th order belong to the Lorentz space $\mathrm {L}_{{p_\circ },1}$, we will denote the space of such mappings by $\mathrm {W}^k_{{p_\circ },1}({\mathbb {R}}^n,{\mathbb {R}}^d)$. We refer to Sect. 2 for relevant definitions and notation.

In this paper, we prove the following Luzin N property with respect to Hausdorff content:

### Theorem 1.1

Let $k\in \{1,\dots ,n\}$,  $q\in [{p_\circ },n]$, and $v\in \mathrm {W}^{k}_{{p_\circ },1}({\mathbb {R}}^n,{\mathbb {R}}^d)$. Then for each $\varepsilon >0$ there exists $\delta >0$ such that for any set $E\subset {\mathbb {R}}^n$ if ${\mathcal {H}}^{q}_\infty (E)<\delta$, then ${\mathcal {H}}_\infty ^{q}(v(E))<\varepsilon$. In particular, ${\mathcal {H}}^{q}(v(E))=0$ whenever ${\mathcal {H}}^{q}(E)=0$.

Here ${\mathcal {H}}^q_\infty (E)$ is as usual the *q*-dimensional Hausdorff content:

$$\begin{aligned} {\mathcal {H}}^q_\infty (E)=\inf \left\{ \sum _{i=1}^\infty (\mathop {\mathrm{diam}}E_i)^q\ : \ E \subset \bigcup \limits _{i=1}^\infty E_i\right\} . \end{aligned}$$

Note that the case $k=1$ was considered in the paper [22], and the case $k>1$, $q>{p_\circ }$ in [24], so we omit them and consider here only the remaining limiting case $q={p_\circ }$, $k>1$.

To study this limiting case, we need a new version of the Sobolev Embedding Theorem that gives inclusions in Lebesgue spaces with respect to suitably general positive measures. For $\beta \in (0,n)$ denote by ${\mathscr {M}}^\beta$ the space of all nonnegative Borel measures $\mu$ on ${\mathbb {R}}^n$ such that

1.1 $$\begin{aligned} | | |\mu | | |_{\beta }=\sup _{I\subset {\mathbb {R}}^n}\ell (I)^{- \beta }\mu (I)<\infty , \end{aligned}$$

where the supremum is taken over all *n*-dimensional cubic intervals $I\subset {\mathbb {R}}^n$ and $\ell (I)$ denotes sidelength. Recall the following classical theorem proved by Adams [2] (see also [5] and [28, Sect. 4.1] ).

### Theorem A

Let $\mu$ be a positive Borel measure on ${\mathbb {R}}^n$ and ${\alpha }>0$, $1<p<q<\infty$, ${\alpha }p<n$. Then for any $f\in \mathrm {L}_p({\mathbb {R}}^n)$ the estimate

1.2 $$\begin{aligned} \int \bigl |I_{\alpha }f|^q\,\mathrm{d}\mu \le C| | |\mu | | |_\beta \cdot \Vert f\Vert ^q_{\mathrm {L}_p} \end{aligned}$$

holds with $\beta =(n-{\alpha }p)\frac{q}{p}$, where *C* depends on $n, \ p, \ q, \ {\alpha }$ only.

Here

$$\begin{aligned} I_{\alpha }f(x)=\int _{{\mathbb {R}}^n}\frac{f(y)}{|y-x|^{n- {\alpha }}}\,\mathrm{d}y \end{aligned}$$

is the Riesz potential of order $\alpha$. The above estimate (1.2) fails for the limiting case $q=p$. Namely, there exist functions $f\in \mathrm {L}_p({\mathbb {R}}^n)$ such that $I_{\alpha }f(x)=+\infty$ on some set of positive $(n-{\alpha }p)$-Hausdorff measure^1^, see for instance [23] and also for further background and history on the question [4]. Nevertheless, we prove the following result for this limiting case $q=p$:

### Theorem 1.2

Let $\mu$ be a positive Borel measure on ${\mathbb {R}}^n$ and ${\alpha }>0$, $1<p<\infty$, ${\alpha }p<n$. Then for any $f\in \mathrm {L}_{p,1}({\mathbb {R}}^n)$ the estimate

1.3 $$\begin{aligned} \Vert I_{\alpha }f\Vert _{L_p(\mu )} \le C| | |\mu | | |^{\frac{1}{p}}_\beta \cdot \Vert f\Vert _{\mathrm {L}_{p,1}}, \end{aligned}$$

holds with $\beta =n-{\alpha }p$, where *C* depends on $n, \ p, \ {\alpha }$ only.

In view of the definition of the Lorentz spaces, it is sufficient to prove the above assertion for the simplest case when *f* coincides with the indicator function of some compact set:

### Theorem 1.3

Let $\mu$ be a positive Borel measure on ${\mathbb {R}}^n$ and ${\alpha }>0$, $1<p<\infty$, ${\alpha }p<n$. Then for any compact set $E\subset {\mathbb {R}}^n$ the estimate

1.4 $$\begin{aligned} \Vert I_{\alpha }(1_E)\Vert ^p_{L_p(\mu )} \le C| | |\mu | | |_\beta \mathop {\mathrm{meas}}(E), \end{aligned}$$

holds with $\beta =n-{\alpha }p$, where $1_E$ is the indicator function of the set *E* and *C* depends on $n, \ p, \ {\alpha }$ only.

Note that our proof of the trace theorem is self-contained, is independent of the previous proofs of these type of results, and uses only very natural and elementary arguments.

From the definition of the space $\mathrm {W}^k_{{p_\circ },1}({\mathbb {R}}^n,{\mathbb {R}}^d)$ of Sobolev–Lorentz mappings and the classical estimate $|\nabla v|\le C|I_{k-1}\nabla ^kv|$, Theorem 1.2 implies

### Theorem 1.4

Let $\mu$ be a positive Borel measure on ${\mathbb {R}}^n$, $k\in \{1,\dots ,n\}$. Then for any function $v\in \mathrm {W}^{k}_{{p_\circ },1}({\mathbb {R}}^n)$ the estimate

1.5 $$\begin{aligned} \int \bigl |\nabla v|^{p_\circ }\,\mathrm{d}\mu \le C| | |\mu | | |_{p_\circ }\cdot \Vert \nabla ^k v\Vert ^{p_\circ }_{\mathrm {L}_{p_\circ },1} \end{aligned}$$

holds, where *C* depends on $n,\ k$ only.

From these results, we deduce also some new differentiability and approximation properties of Sobolev–Lorentz mappings $v\in \mathrm {W}^{k}_{{p_\circ },1}({\mathbb {R}}^n)$. Namely, for $m\le n$ the *m*-order derivatives $\nabla ^m v$ are well-defined ${\mathcal {H}}^{m{p_\circ }}$–almost everywhere, a function *v* is *m*-times differentiable (in the classical Fréchet–Peano sense) ${\mathcal {H}}^{m{p_\circ }}$–almost everywhere, and, finally, it coincides with $C^m$-smooth function on ${\mathbb {R}}^n\setminus U$, where the open exceptional set *U* has small ${\mathcal {H}}^{m{p_\circ }}_\infty$-Hausdorff content (see Theorems 3.9, 3.11–3.12 ). Note that for mappings of the classical Sobolev space $\mathrm {W}^{k}_{{p_\circ }}({\mathbb {R}}^n)$, the corresponding exceptional set *U* has small Bessel capacity ${\mathcal {B}}_{k-m,p}(U)<\varepsilon$, and, respectively, the gradients $\nabla ^m v$ are well defined in ${\mathbb {R}}^n$ except for some exceptional set of zero Bessel capacity ${\mathcal {B}}_{k-m,p}$ (see, e.g., [9] and Chap. 3 in [36]).

In the last Subsect. 3.5, we discuss Morse–Sard-type theorems for Sobolev–Lorentz mappings. Namely, for an open set $\Omega \subset {\mathbb {R}}^n$ and a mapping $v\in \mathrm {W}^{k}_{{p_\circ }, 1, {\mathrm{loc}}}(\Omega ,{\mathbb {R}}^d)$ denote $Z_{v,m}=\{x\in \Omega : v$ is differentiable at *x* and $\mathop {\mathrm{rank}}\nabla v(x)<m\}$ (recall, that by previous results *v* is differentiable ${\mathcal {H}}^{p_\circ }$ a.e.). We state:

### Theorem 1.5

If $k,m\in \{1,\dots ,n\}$, $\Omega$ is an open subset of ${\mathbb {R}}^n$, and $v\in \mathrm {W}^{k}_{{p_\circ },1,{\mathrm{loc}}}(\Omega ,{\mathbb {R}}^d)$, then ${\mathcal {H}}^{q_\circ }(v(Z_{v,m}))=0$.

Here

1.6 $$\begin{aligned} {p_\circ }=\frac{n}{k} \quad \text{ and } \quad {q_\circ }=m-1+\frac{n-m+1}{k}={p_\circ }+(m- 1)\bigl (1-k^{-1}\bigr ). \end{aligned}$$

The theorem was proved for $\mathrm {C}^k$-smooth functions by Morse [30] in 1939 for the case $k=n$, $m=d={q_\circ }=1$, and subsequently by Sard [32] in 1942 for $k=n-m+1$, $m=d={q_\circ }$. For arbitrary natural values *k*, *n*, *m*, and $\mathrm {C}^k$-smooth functions, the result was proved almost simultaneously by Dubovitskiĭ [15] in 1967 and Federer [18, Theorem 3.4.3] in 1969^2^.

The Morse–Sard Theorem for Sobolev spaces $W^k_p({\mathbb {R}}^n,{\mathbb {R}}^m)$ with $p>n$ (i.e., when $W^k_p({\mathbb {R}}^n)\hookrightarrow C^{k-1}({\mathbb {R}}^n)$ ) was obtained in [13] (see also [20] for a simple proof), and for Lipschitz and Hölder continuous mappings $\mathrm {C}^{k,\lambda }$ — see, e.g., in [7, 8], respectively. More facts about history of this issue could be found in our papers [11, 12, 24], where the above Theorem 1.5 was proved in the Sobolev context $\mathrm {W}^k_{p_\circ }({\mathbb {R}}^n)$ for $k,m\in \{2,\dots ,n\}$. For $k=1$ (i.e., ${q_\circ }=n$) it is folklore (see, e.g., [33]), so in the present paper, we need to only consider the case $m=1$, ${q_\circ }={p_\circ }=\frac{n}{k}$.

Let us remark, in conclusion, that an interesting phenomenon occurs for functions of the Sobolev–Lorentz space $W^k_{{p_\circ },1}({\mathbb {R}}^n,{\mathbb {R}}^d)$. On the one hand, the order of integrability is very sharp—the minimal order, that guaranties a priori only **continuity** of mappings. On the other hand, these mappings a posteriori have many additional analytical regularity properties: the Luzin *N*-property, differentiability and approximation properties, and the Morse–Sard property (see above).

For instance, if $k=n-m+1$, then almost all level sets of mappings $v\in \mathrm {W}^k_{{p_\circ },1}({\mathbb {R}}^n,{\mathbb {R}}^m)$ are $\mathrm {C}^1$-smooth manifolds [24]. (The result should be contrasted with the fact that mappings of class $\mathrm {W}^k_{{p_\circ },1}({\mathbb {R}}^n,{\mathbb {R}}^m)$ are continuous only and need not to be $\mathrm {C}^1$-smooth in general. ) This property recently found some applications in mathematical fluid mechanics (see [25]).

## Preliminaries

By an *n* *-dimensional cubic interval*, we mean a closed cube in ${\mathbb {R}}^n$ with sides parallel to the coordinate axes. If *Q* is an *n*-dimensional cubic interval, then we write $\ell (Q)$ for its sidelength.

For a subset *S* of ${\mathbb {R}}^n$, we write ${\mathscr {L}}^n(S)$ for its outer Lebesgue measure. The *m*-dimensional Hausdorff measure is denoted by ${\mathcal {H}}^m$ and the *m*-dimensional Hausdorff content by ${\mathcal {H}}^{m}_{\infty }$. Recall that for any subset *S* of ${\mathbb {R}}^n$ we have by definition

$$\begin{aligned} {\mathcal {H}}^m (S)=\lim \limits _{\alpha \searrow 0}{\mathcal {H}}^m_\alpha (S) = \sup _{\alpha >0} {\mathcal {H}}^{m}_{\alpha }(S), \end{aligned}$$

where for each $0< \alpha \le \infty$,

$$\begin{aligned} {\mathcal {H}}^m_\alpha (S)=\inf \left\{ \sum _{i=1}^\infty (\mathop {\mathrm{diam}}S_i)^m\ :\ \mathop {\mathrm{diam}}S_i\le \alpha ,\ \ S \subset \bigcup \limits _{i=1}^\infty S_i \right\} . \end{aligned}$$

It is well known that ${\mathcal {H}}^n(S) = {\mathcal {H}}^n_\infty (S)\sim {\mathscr {L}}^n(S)$ for sets $S\subset {\mathbb {R}}^n$.

To simplify the notation, we write $\Vert f\Vert _{\mathrm {L}_p}$ instead of $\Vert f\Vert _{\mathrm {L}_p({\mathbb {R}}^n)}$, etc.

The Sobolev space $\mathrm {W}^{k}_{p} ({\mathbb {R}}^n,{\mathbb {R}}^d)$ is as usual defined as consisting of those ${\mathbb {R}}^d$-valued functions $f\in \mathrm {L}_p({\mathbb {R}}^n)$ whose distributional partial derivatives of orders $l\le k$ belong to $\mathrm {L}_p({\mathbb {R}}^n)$ (for detailed definitions and differentiability properties of such functions see, e.g., [14, 16, 28, 36]). Denote by $\nabla ^{l} f$ the vector-valued function consisting of all *l*-th order partial derivatives of *f* arranged in some fixed order. However, for the case of first-order derivatives $l=1$, we shall often think of $\nabla f(x)$ as the Jacobi matrix of *f* at *x*, thus the $d \times n$ matrix whose *r*-th row is the vector of partial derivatives of the *r*-th coordinate function.

We use the norm

$$\begin{aligned} \Vert f\Vert _{\mathrm {W}^{k}_{p}}=\Vert f\Vert _{\mathrm {L}_p}+\Vert \nabla f\Vert _{\mathrm {L}_p}+\dots +\Vert \nabla ^kf\Vert _{\mathrm {L}_p}, \end{aligned}$$

and unless otherwise specified all norms on the spaces ${\mathbb {R}}^s$ ($s \in {\mathbb {N}}$) will be the usual euclidean norms.

Working with locally integrable functions, we always assume that the precise representatives are chosen. If $w\in L_{1,\mathrm{loc}}(\Omega )$, then the precise representative $w^*$ is defined for *all* $x \in \Omega$ by2.1where the dashed integral as usual denotes the integral mean,and $B(x,r)=\{y: |y-x|<r\}$ is the open ball of radius *r* centered at *x*. Henceforth, we omit special notation for the precise representative writing simply $w^* = w$.

We will say that *x* is an $\mathrm {L}_p$-Lebesgue point of *w* (and simply a Lebesgue point when $p=1$), ifIf $k<n$, then it is well known that functions from Sobolev spaces $\mathrm {W}^{k}_{p}({\mathbb {R}}^n)$ are continuous for $p>\frac{n}{k}$ and could be discontinuous for $p\le {p_\circ }=\frac{n}{k}$ (see, e.g., [28, 36]). The Sobolev–Lorentz space $\mathrm {W}^{k}_{{p_\circ },1}({\mathbb {R}}^n)\subset \mathrm {W}^{k}_{{p_\circ }}({\mathbb {R}}^n)$ is a refinement of the corresponding Sobolev space that for our purposes turns out to be convenient. Among other things functions that are locally in $\mathrm {W}^{k}_{{p_\circ },1}$ on ${\mathbb {R}}^n$ are in particular continuous.

Given a measurable function $f:{\mathbb {R}}^n\rightarrow {\mathbb {R}}$, denote by $f_{*}:(0,\infty ) \rightarrow {\mathbb {R}}$ its distribution function

$$\begin{aligned} f_{*}(s) := {\mathscr {L}}^n\left\{ x\in {\mathbb {R}}^n: \, |f(x)|> s \right\} , \end{aligned}$$

and by $f^*$ the nonincreasing rearrangement of *f*, defined for $t>0$ by

$$\begin{aligned} f^*(t)=\inf \left\{ s \ge 0 :\, f_{*}(s) \le t\right\} . \end{aligned}$$

Since |*f*| and $f^*$ are equimeasurable, we have for every $1\le p<\infty$,

$$\begin{aligned} \biggl (\int _{{\mathbb {R}}^n}|f(x)|^p\, \mathrm{d}x\biggr )^{1/p}=\biggr (\int _0^{+\infty }f^*(t)^p\, \mathrm{d}t\biggr )^{1/p}. \end{aligned}$$

The Lorentz space $\mathrm {L}_{p,q}({\mathbb {R}}^n)$ for $1\le p<\infty$, $1\le q<\infty$ can be defined as the set of all measurable functions $f:{\mathbb {R}}^n\rightarrow {\mathbb {R}}$ for which the expression

$$\begin{aligned} \Vert f\Vert _{\mathrm {L}_{p,q}}= \left\{ \begin{array}{ll} {\displaystyle \biggl (\frac{q}{p} \int _0^{+\infty }(t^{1/p}f^*(t))^q\frac{\mathrm{d}t}{t}\biggr )^{1/q}} &{} {\displaystyle \quad \text{ if } \quad 1 \le q < \infty }\\ {\displaystyle \sup _{t>0}t^{1/p}f^*(t)} &{} {\displaystyle \quad \text{ if } \quad q = \infty } \end{array} \right. \end{aligned}$$

is finite. We refer the reader to [27, 35, 36] for information about Lorentz spaces. However, let us remark that in view of the definition of $\Vert \cdot \Vert _{\mathrm {L}_{p,q}}$ and the equimeasurability of *f* and $f^*$ we have $\Vert f \Vert _{\mathrm {L}_p} = \Vert f \Vert _{\mathrm {L}_{p,p}}$ so that in particular $\mathrm {L}_{p,p}({\mathbb {R}}^n )=\mathrm {L}_{p}({\mathbb {R}}^n )$. Further, for a fixed exponent *p* and $q_1 < q_2$, we have the estimate $\Vert f\Vert _{\mathrm {L}_{p,q_2}}\le \Vert f\Vert _{\mathrm {L}_{p,q_1}}$, and, consequently, the embedding $\mathrm {L}_{p,q_1}({\mathbb {R}}^n ) \subset \mathrm {L}_{p,q_2}({\mathbb {R}}^n )$ (see [27, Theorem 3.8(a)]). Finally, we recall that $\Vert \cdot \Vert _{\mathrm {L}_{p,q}}$ is a norm on $\mathrm {L}_{p,q}({\mathbb {R}}^n )$ for all $q \in [1,p]$ and a quasi-norm in the remaining cases $q \in (p,\infty ]$ (see [27, Proposition 3.3]).

Here we shall mainly be concerned with the Lorentz space $\mathrm {L}_{p,1}$, and in this case, one may rewrite the norm as (see for instance [27, Proposition 3.6])

2.2 $$\begin{aligned} \Vert f\Vert _{p,1}= \int _0^{+\infty }\bigl [{\mathscr {L}}^n(\{x\in {\mathbb {R}}^n:|f(x)|>t\})\bigr ]^{ \frac{1}{p}} \, \mathrm{d}t. \end{aligned}$$

We record the following subadditivity property of the Lorentz norm for later use.

### Lemma 2.1

(see, e.g., [27, 31]) Suppose that $1\le p<\infty$ and $E=\bigcup _{j \in {\mathbb {N}}} E_j$, where $E_j$ are measurable and mutually disjoint subsets of ${\mathbb {R}}^n$. Then for all $f\in \mathrm {L}_{p,1}$ we have

$$\begin{aligned} \sum _j\Vert f\cdot 1_{E_j}\Vert ^p_{\mathrm {L}_{p,1}}\le \Vert f\cdot 1_E\Vert ^p_{\mathrm {L}_{p,1}}, \end{aligned}$$

where $1_E$ denotes the indicator function of the set *E*.

Denote by $\mathrm {W}^{k}_{p,1}({\mathbb {R}}^n)$ the space of all functions $v\in \mathrm {W}^k_p({\mathbb {R}}^n)$ such that in addition the Lorentz norm $\Vert \nabla ^k v\Vert _{\mathrm {L}_{p,1}}$ is finite.

For a mapping $$u \in \mathrm {L}_1(Q,{\mathbb {R}}^d)$$, $$Q\subset {\mathbb {R}}^n$$, $$m\in {\mathbb {N}}$$, define the polynomial $$P_{Q,m}[u] $$ of degree at most *m* by the following rule:

2.3 $$\begin{aligned} \int _Qy^\alpha \left( u(y)-P_{Q,m}[u](y) \right) \,\mathrm{d}y=0 \end{aligned}$$

for any multi-index $\alpha =(\alpha _1,\dots ,\alpha _n)$ of length $|\alpha |=\alpha _1+\dots +\alpha _n\le {m}$. Denote $P_{Q}[u]= P_{Q,k-1}[u]$.

The following well-known bound will be used on several occasions.

### Lemma 2.2

Suppose $v\in \mathrm {W}^{k}_{{p_\circ },1}({\mathbb {R}}^n,{\mathbb {R}}^d)$ with $k\in \{1,\dots ,n\}$. Then *v* is a continuous mapping and for any *n*-dimensional cubic interval $Q\subset {\mathbb {R}}^n$ the estimate

2.4 $$\begin{aligned} \sup \limits _{y\in Q}\bigl |v(y)-P_{Q}[v](y)\bigr |\le C\Vert 1_Q\cdot \nabla ^{k} v\Vert _{\mathrm {L}_{{p_\circ },1}} \end{aligned}$$

holds, where *C* is a constant depending on *n*, *d* only. Moreover, the mapping $v_{Q}(y)=v(y)-P_{Q}[v](y)$, $y\in Q$, can be extended from *Q* to the whole of ${\mathbb {R}}^n$ such that the extension (denoted again) $v_{Q}\in \mathrm {W}^{k}_{{p_\circ },1}({\mathbb {R}}^n,{\mathbb {R}}^d)$ and

2.5 $$\begin{aligned} \Vert \nabla ^{k} v_{Q}\Vert _{\mathrm {L}_{{p_\circ },1}({\mathbb {R}}^n)}\le C_0 \Vert \nabla ^{k} v\Vert _{\mathrm {L}_{{p_\circ },1}(Q)}, \end{aligned}$$

where $C_0$ also depends on *n*, *d* only.

### Proof

For continuity and the estimate (2.4) see [24, Lemma 1.3]. Because of coordinate invariance of estimate (2.5), it is sufficient to prove the assertions about extension for the case when *Q* is a unit cube: $Q=[0,1]^n$. Put $u(y)= v(y)-P_{Q}[v](y)$ for $y\in Q$.

By Peetre theorem (see Theorem 6.5 in [27, p. 10]), it is easy to deduce that

2.6 $$\begin{aligned} \Vert \nabla ^m u\Vert _{L_{{p_\circ },1}(Q)}\le C \Vert \nabla ^k u\Vert _{L_{{p_\circ },1}(Q)}\qquad \forall m=0,1,\dots ,k-1. \end{aligned}$$

Using the standard Extension operator for Sobolev spaces (the well-known *finite-order reflection* procedure, see, e.g., [28, Sect. 1.1.17]), function *u* on the unit cube $Q=[0,1]^n$ can be extended to a function $U \in \mathrm {W}^{k}_{{p_\circ },1}({\mathbb {R}}^n)$ such that the estimate

$$\begin{aligned} \Vert \nabla ^{k} U\Vert _{\mathrm {L}_{{p_\circ },1}({\mathbb {R}}^n)}\le C'\sum \limits _{m=0}^k \Vert \nabla ^m u\Vert _{L_{{p_\circ },1}(Q)} \end{aligned}$$

holds. Taking into account the identity $\nabla ^k u\equiv \nabla ^k v$ on *Q* and (2.6), we obtain the required estimate (2.5). $\square$

### Corollary 2.3

(see, e.g., [24]) Suppose $v\in \mathrm {W}^{k}_{{p_\circ },1}({\mathbb {R}}^n,{\mathbb {R}}^d)$ with $k\in \{1,\dots ,n\}$. Then *v* is a continuous mapping and for any *n*-dimensional cubic interval $Q\subset {\mathbb {R}}^n$ the estimate

2.7 $$\begin{aligned} \mathop {\mathrm{diam}}v(Q)\le C\biggl (\frac{\Vert \nabla v\Vert _{\mathrm {L}_1(Q)}}{\ell (Q)^{n-1}}+ \Vert 1_Q\cdot \nabla ^{k} v\Vert _{\mathrm {L}_{{p_\circ },1}}\biggr )\le C\biggl (\frac{\Vert \nabla v\Vert _{\mathrm {L}_{p_\circ }(Q)}}{\ell (Q)^{k-1}}+ \Vert 1_Q\cdot \nabla ^{k} v\Vert _{\mathrm {L}_{{p_\circ },1}}\biggr ) \end{aligned}$$

holds.

The above results can easily be adapted to give that $v \in \mathrm {C}_{0}({\mathbb {R}}^n )$, the space of continuous functions on ${\mathbb {R}}^n$ that vanish at infinity (see for instance [27, Theorem 5.5]).

Analogously, from previous estimates one could deduce

### Corollary 2.4

Suppose $v\in \mathrm {W}^{k}_{{p_\circ },1}({\mathbb {R}}^n,{\mathbb {R}}^d)$ with $k\in \{1,\dots ,n\}$. Then for all $m\in \{1,\dots ,k\}$ and for any *n*-dimensional cubic interval $Q\subset {\mathbb {R}}^n$ the estimate

2.8 $$\begin{aligned} \sup \limits _{y\in Q}\bigl |v(y)-P_{Q,m-1}[v](y)\bigr |\le C\biggl (\frac{\Vert \nabla ^m v\Vert _{\mathrm {L}_{p_\circ }(Q)}}{\ell (Q)^{k-m}}+ \Vert 1_Q\cdot \nabla ^{k} v\Vert _{\mathrm {L}_{{p_\circ },1}}\biggr ) \end{aligned}$$

holds.

Here, we list some standard facts about the Lorentz spaces.

### Theorem 2.5

(Boundedness of the maximal operator, see [27]) Let $f\in \mathrm {L}_{p,q}({\mathbb {R}}^n)$,  $1< p < \infty$,  $1\le q < \infty$. Then

$$\begin{aligned} \Vert {\mathcal {M}}f\Vert _{\mathrm {L}_{p,q}}\le C\Vert f\Vert _{\mathrm {L}_{p,q}}. \end{aligned}$$

Here

$$\begin{aligned} {\mathcal {M}}f(x)=\sup \limits _{r>0}\,r^{-n}\int _{B(x,r)}|f(y)|\,\mathrm{d}y \end{aligned}$$

is the usual Hardy–Littlewood maximal function of *f*.

### Corollary 2.6

(Regularization in Lorentz spaces [27]) Let  $1< p < \infty$,  $1\le q < \infty$. Suppose that $f\in \mathrm {L}_{p,q}({\mathbb {R}}^n)$ and $\psi \in \mathrm {C}_0^\infty ({\mathbb {R}}^n)$ is a standard mollifier. Then $\psi _\delta *f\rightarrow f$ in $\mathrm {L}_{p,q}({\mathbb {R}}^n)$ as $\delta \rightarrow 0$.

Here and henceforth $\mathrm {C}_0^\infty ({\mathbb {R}}^n)$ means the space of $\mathrm {C}^\infty$ smooth and compactly supported functions on ${\mathbb {R}}^n$.

### Corollary 2.7

(Regularization in Sobolev–Lorentz spaces) If $f\in \mathrm {W}^k_{p,q}({\mathbb {R}}^n)$,  $1< p < \infty$,  $1\le q < \infty$, then there exists a sequence of smooth functions $f_i\in \mathrm {C}_0^\infty ({\mathbb {R}}^n)$ such that $\Vert \nabla ^m(f-f_i)\Vert _{\mathrm {L}_p({\mathbb {R}}^n)}\rightarrow 0$ for $m=0,1,\dots ,k$, $\Vert \nabla ^k(f-f_i)\Vert _{\mathrm {L}_{p,q}({\mathbb {R}}^n)}\rightarrow 0$ as $i\rightarrow \infty$.

### Remark 2.8

By Sobolev inequality, under conditions of Corollary 2.7, if, in addition $1\le q\le p$ and $(k-m)p<n$ for some $m\in \{0,1,\dots ,k-1\}$, then we have also the convergence $\Vert \nabla ^m(f-f_i)\Vert _{L_{p_m,q}({\mathbb {R}}^n)}\rightarrow 0$ , where $p_m$ is a Sobolev exponent $p_m=\frac{np}{n-(k-m)p}$ (see, e.g., [27, Sect. 8] ).

We need also the following important Adams strong-type estimates for maximal functions.

### Theorem 2.9

(see Theorem A, Proposition 1 and its Corollary in [1]) Let $\beta \in (0,n)$. Then for nonnegative functions $f\in \mathrm {C}_0({\mathbb {R}}^n)$ the estimates

$$\begin{aligned} \int _0^\infty {\mathcal {H}}^{\beta }_\infty \left( \{x\in {\mathbb {R}}^n : {\mathcal {M}}f(x)\ge t \}\right) \,\mathrm{d}t\le & {} C_1 \int _0^\infty {\mathcal {H}}^{\beta }_\infty \left( \{x\in {\mathbb {R}}^n : f(x)\ge t \}\right) \,\mathrm{d}t\\\le & {} C_2\sup \biggl \{\int f\,\mathrm{d}\mu :\mu \in {\mathscr {M}}^\beta ,\ | | |\mu | | |_\beta \le 1\biggr \}, \end{aligned}$$

hold, where the constants $C_1,C_2$ depend on $\beta ,n$ only.

We need also the following classical fact (cf. with [10] ).

### Lemma 2.10

(see Lemma 2 in [14]) Let $u\in \mathrm {W}^{m}_1({\mathbb {R}}^n)$, $m\le n$. Then for any *n*-dimensional cubic interval $Q\subset {\mathbb {R}}^n$, $x\in Q$, and for any $j=0,1,\dots ,m-1$ the estimate

2.9 $$\begin{aligned} \bigl |\nabla ^ju(x)-\nabla ^jP_{Q,m-1}[u](x)\bigr |\le C\ell (Q)^{m-j}({\mathcal {M}}\nabla ^m u)(x) \end{aligned}$$

holds, where the constant *C* depends on *n*, *m* only.

## Proofs of the Main Results

### The Trace Theorem

Theorem 1.3 plays the key role among other results. Its proof splits into a number of lemmas. Fix parameters $m>0$, $1<p<\infty$, $0<\alpha p<n$, and a positive Borel measure $\mu$ on ${\mathbb {R}}^n$ satisfying

3.1 $$\begin{aligned} \mu (B(x,r))\le r^{n-\alpha p} \end{aligned}$$

for every ball $B(x,r)\subset {\mathbb {R}}^n$. Fix also a compact set $E\subset {\mathbb {R}}^n$. Denote by $I_E$ the corresponding Riesz potential $I_\alpha (1_E)$.

It is very easy to check by standard calculation that

3.2 $$\begin{aligned} 0\le I_E(x)\le C_0 |E|^{\frac{\alpha }{n}}, \end{aligned}$$

where the constant $C_0$ depends on $n,\alpha$ only.

Denote also $t_m=2^m$ (here $m\in {\mathbb {Z}}$),

$$\begin{aligned} E_m= & {} \{x\in E:I_E(x)\in [t_m,2t_m]\},\\ E'_{m}= & {} \{x\in E:I_E(x)\le t_m\},\qquad E''_m=\{x\in E:I_E(x)>t_m\}. \end{aligned}$$

In this section, we will write $f\lesssim g$, if $f\le C g$, where *C* depends on $n,\alpha ,p$ only (really, most of the corresponding constants below up to Lemma 3.6 depends on $n,\alpha$ only).

#### Lemma 3.1

There exists a positive constant $m_0\in {\mathbb {N}}$ depending on $n,\alpha$ only such that for any $m\in {\mathbb {Z}}$ and $x\in {\mathbb {R}}^n$  if $I_E(x)\ge t_m$, then $I_{E''_{m-m_0}}(x)\gtrsim t_m$.

#### Proof

The claim follows from the well-known maximum principle: $I_{E'_m}(x)\le 2^{n-\alpha }t_m$ for every $m\in {\mathbb {Z}}$ (see [21, Theorem 5.2]). $\square$

#### Lemma 3.2

For any $x,y\in {\mathbb {R}}^n$ if $I_E(y){\ge } t$ and $|x-y|\le (2t)^{\frac{1}{\alpha }}$ then $I_E(x)\gtrsim t$.

#### Proof

Let $$I_E(y){\ge } t$$ and

3.3 $$\begin{aligned} |y-x|\le (2t)^{\frac{1}{\alpha }}. \end{aligned}$$

Denote $r=|y-x|$, $B=B(y,r)=\{z\in {\mathbb {R}}^n:|z-y|<r\}$. Then by construction

3.4 $$\begin{aligned} t \, {\le } \, I_E(y)=I_{E\cap B}(y)+I_{E\setminus B}(y). \end{aligned}$$

Consider two possible situations.

(I) $I_{E\cap B}(y)\le \frac{t}{2}$, then $I_{E\setminus B}(y)\ge \frac{t}{2}$. For any $z\in E\setminus B$ we have $|z-y|\ge r=|x-y|$, thus, $|x-z|\le |x-y|+|z-y|\le 2|z-y|$, consequently,

3.5 $$\begin{aligned} I_{E}(x)\ge I_{E\setminus B}(x)\ge 2^{n-\alpha }I_{E\setminus B}(y)\ge 2^{n-\alpha -1}t. \end{aligned}$$

(II) $I_{E\cap B}(y)\ge \frac{t}{2}$. Then (3.2) implies $\frac{t}{2}\le C_0|B\cap E|^{\frac{\alpha }{n}}$. Since $B\cap E\subset B(x,2r)$, by elementary estimates we have

$$\begin{aligned} I_E(x)\ge \frac{|B\cap E|}{(2r)^{n-\alpha }}\ge C'\frac{t^{\frac{n}{{\alpha }}}}{r^{n-\alpha }} \overset{{(3.3)}}{\ge }C''\frac{t^{\frac{n}{{\alpha }}}}{t^{\frac{n}{{\alpha }}-1}}=C_2t. \end{aligned}$$

$$\square $$

Denote $F_m=\{x\in {\mathbb {R}}^n:I_E(x)\in [t_m,2t_m]\}$, $\mu _m=\mu (F_m)$, $\mu _m(\cdot )=\mu \,\llcorner \,F_m$. By construction,

3.6 $$\begin{aligned} \Vert I_\alpha (1_E)\Vert ^p_{L_p(\mu )}\sim \sum \limits _{m=-\infty }^\infty t_m^p\mu _m. \end{aligned}$$

So our main purpose below is to estimate $t_m\mu _m$. Of course, $t_m\mu _m\le \int _{{\mathbb {R}}^n}I_E(x)\,\mathrm{d}\mu _m(x)$. By Fubini’s Theorem, we have

3.7 $$\begin{aligned} \int _{{\mathbb {R}}^n}I_{E}(x)\,\mathrm{d}\mu _m(x)= & {} \int _0^\infty \rho ^{-n+\alpha -1}\left[ \ \int _{{\mathbb {R}}^n}|E\cap B(x,\rho )|\, \mathrm{d}\mu _m(x)\right] \mathrm{d}\rho \nonumber \\= & {} \int _0^\infty \rho ^{-n+\alpha -1}\left[ \ \int _{E}\mu _m\bigl [B(y,\rho )\bigr ]\,\mathrm{d}y\right] \mathrm{d}\rho . \end{aligned}$$

#### Lemma 3.3

The estimate

3.8 $$\begin{aligned} t_m\mu _m \lesssim \int _0^\infty \rho ^{-n+\alpha -1}\left[ \ \int _{{\mathbb {R}}^n}|E''_{m-m_0}\cap B(x,\rho )|\, \mathrm{d}\mu _m(x)\right] \mathrm{d}\rho \end{aligned}$$

holds, where $m_0$ is a constant from Lemma 3.1.

#### Proof

By Lemma 3.1, $I_{E''_{m-m_0}}\ge C_1 t_m$ on $F_m$, therefore $t_m\mu _m\le C\int _{{\mathbb {R}}^n}I_{E''_{m-m_0}}(x)\, \mathrm{d}\mu _m(x)$, and the last inequality implies in conjunction with Fubini’s Theorem (3.7). $\square$

#### Lemma 3.4

There exists a constant $m_1\in {\mathbb {N}}$ such that

3.9 $$\begin{aligned} t_m\mu _m\lesssim \int _{t_{m-m_1}^{\frac{1}{{\alpha }}}}^\infty \rho ^{-n+\alpha -1}\left[ \ \int _{{\mathbb {R}}^n}|E''_{m-m_0}\cap B(x,\rho )|\,\mathrm{d}\mu _m(x)\right] \mathrm{d}\rho . \end{aligned}$$

#### Proof

Let $m_1\in {\mathbb {N}}$, its exact value will be specified below. We have $|E\cap B(x,\rho )|\le \omega _n\rho ^n$, where $\omega _n$ is a volume of a unit ball in ${\mathbb {R}}^n$. Thus

$$\begin{aligned}&\int _0^{t_{m-m_1}^{\frac{1}{{\alpha }}}}\rho ^{-n+\alpha -1}\left[ \ \int _{{\mathbb {R}}^n}|E\cap B(x,\rho )|\,d\mu _m(x)\right] d\rho \\&\quad \le \omega _n \mu _m\int _0^{t_{m-m_1}^{\frac{1}{{\alpha }}}}\rho ^{\alpha -1}\,d\rho =\frac{\omega _n}{{\alpha }}\mu _m t_{m-m_1}=\frac{\omega _n}{{\alpha }}2^{-m_1}\mu _m t_m. \end{aligned}$$

So the target estimate (3.9) follows from (3.8) provided that $\frac{1}{{\alpha }}\omega _n2^{-m_1}$ is sufficiently small. $\square$

#### Lemma 3.5

There exists a constant $i_0\in {\mathbb {N}}$ such that for all $i\ge m-m_1$ the equality

3.10 $$\begin{aligned}&\int _{t_{i}^{\frac{1}{{\alpha }}}}^{t_{i+1}^{\frac{1}{{\alpha }}}}\rho ^{-n+\alpha -1}\left[ \ \int _{{\mathbb {R}}^n}|E''_{m-m_0}\cap B(x,\rho )|\,\mathrm{d}\mu _m(x)\right] \mathrm{d}\rho \nonumber \\&\quad = \sum \limits _{j=m-m_0}^{i+i_0} \int _{t_{i}^{\frac{1}{{\alpha }}}}^{t_{i+1}^{\frac{1}{{\alpha }}}}\rho ^{-n+\alpha -1}\left[ \ \int _{{\mathbb {R}}^n}|E_j\cap B(x,\rho )|\,\mathrm{d}\mu _m(x)\right] \mathrm{d}\rho \end{aligned}$$

holds, where $m_0,\,m_1$ are the constants from Lemmas 3.1 and 3.4, respectively.

#### Proof

Let $$i\ge m-m_1$$,

3.11 $$\begin{aligned} \rho ^\alpha \le t_{i+1}, \end{aligned}$$

and $x\in F_m=\mathop {\mathrm{supp}}\mu _m$. Then by definitions

3.12 $$\begin{aligned} I_E(x)\le 2t_m. \end{aligned}$$

Take any point $y\in E''_{m-m_0}\cap B(x,\rho )$, then by construction $y\in E_j$ for some index $j\ge m-m_0$, in particular, $I_E(y)\ge t_j$. Suppose $j\ge i+1$. Then (3.11) implies $|x-y|^{\alpha }\le t_{i+1}\le t_j$, therefore, by Lemma 3.2 (applying for $t=t_j$) we have $I_E(x)\ge C_2t_j$. Thus by (3.12) we obtain $j\le m+m_2$ for some constant $m_2$ depending on $\alpha ,\,n$ only.

Finally, we have $j\le \max (i+1,m+m_2)\le \max (i+1,i+m_1+m_2)$ finishing the proof of the Lemma. $\square$

#### Lemma 3.6

The estimate

3.13 $$\begin{aligned} t_m\mu _m\lesssim \sum \limits _{j=m-m_0}^\infty |E_j|t_{j-i_0}^{1-p} \end{aligned}$$

holds for all $m\in {\mathbb {Z}}$, where $m_0$, $i_0$ are the constants from Lemmas 3.1 and 3.5, respectively.

#### Proof

We have3.14where the symbol ${\ i\leftrightarrow j}$ indicates that the order of summation was changed. $\square$

#### Lemma 3.7

The estimate

3.15 $$\begin{aligned} \sum \limits _{m=-\infty }^\infty t^p_m\mu _m\lesssim |E| \end{aligned}$$

holds.

#### Proof

We have3.16where again the symbol ${\ m\leftrightarrow j}$ means that the order of summation was changed. $\square$

Combining Lemma 3.7 with the initial estimate (3.6) gives the validity of the Trace Theorem 1.3.

### On Approximation of Sobolev–Lorentz Mappings

Using the established Theorem 1.2 and Adam’s estimate from Theorem 2.9 with $\beta =n-(k-l)p$, we obtain the following estimates, which are key ingredients in the proof of *N*-property.

#### Corollary 3.8

Let $p\in (1, \infty )$, $k,l\in \{1,\dots ,n\}$, $l\le k$, $(k-l)p<n$. Then for any function $f\in \mathrm {W}^{k}_{p,1}({\mathbb {R}}^n)$ the estimates

3.17 $$\begin{aligned}&\Vert \nabla ^l f\Vert ^p_{L_p(\mu )} \le C| | |\mu | | |_\beta \Vert \nabla ^k f\Vert ^p_{\mathrm {L}_{p,1}}\quad \forall \mu \in {\mathscr {M}}^\beta , \end{aligned}$$

3.18 $$\begin{aligned}&\int _0^\infty {\mathcal {H}}^{\beta }_\infty \left( \left\{ x\in {\mathbb {R}}^n : {\mathcal {M}}\bigl (|\nabla ^l f|^p\bigr )(x)\ge t \right\} \right) \,\mathrm{d}t \le C\Vert \nabla ^k f\Vert ^p_{\mathrm {L}_{p,1}} \end{aligned}$$

hold, where $\beta =n-(k-l)p$ and the constant *C* depends on *n*, *k*, *p* only.

The main result of this subsection is the following

#### Theorem 3.9

Let $p\in (1, \infty )$, $k,l\in \{1,\dots ,n\}$, $l\le k$, $(k-l)p<n$. Then for any $f\in \mathrm {W}^{k}_{p,1}({\mathbb {R}}^n)$ and for each $\varepsilon >0$ there exist an open set $U\subset {\mathbb {R}}^n$ and a function $g\in \mathrm {C}^l({\mathbb {R}}^n)$ such that

(i)  $${\mathcal {H}}^{n-(k-l)p}_\infty (U)<\varepsilon $$;
(ii)  each point $x\in {\mathbb {R}}^n\setminus U$ is an $\mathrm {L}_{p}$-Lebesgue point for $\nabla ^j f$,  $j=0,\dots ,l$;
(iii)  $f\equiv g$, $\nabla ^jf \equiv \nabla ^jg$ on ${\mathbb {R}}^n\setminus U$ for $j=1,\dots ,l$.

Note that in the analogous theorem for the case of Sobolev mappings $f\in \mathrm {W}^{k}_{p}({\mathbb {R}}^n)$, the assertion (i) should be reformulated as follows:

(i’) $\mathcal B_{k-l,p}(U)<\varepsilon$ if $l<k$, where $\mathcal B_{\alpha ,p}(U)$ denotes the Bessel capacity of the set *U* (see, e.g., [36, Chap. 3] or [9] ).

Recall that for $1<p<\infty$ and $0<n - \alpha p <n$, the smallness of ${\mathcal {H}}^{n - \alpha p}_\infty (U)$ implies the smallness of ${\mathcal {B}}_{\alpha ,p}(U)$, but the opposite false since ${\mathcal {B}}_{\alpha ,p}(U)=0$ whenever ${\mathcal {H}}^{n - \alpha p}(U)<\infty$. On the other hand, for $1<p<\infty$ and $0<n - \alpha p < \beta \le n$, the smallness of ${\mathcal {B}}_{\alpha ,p}(U)$ implies the smallness of ${\mathcal {H}}^\beta _\infty (U)$ (see, e.g., [6]). So the usual assertion (i’) is essentially weaker than (i).

#### Proof of Theorem 3.9

Let the assumptions of the Theorem be fulfilled. By Theorem 2.5 and Corollary 2.7, we can choose the sequence of mappings $f_i\in C_0^\infty ({\mathbb {R}}^n)$ such that $\Vert \nabla ^kf-\nabla ^kf_i\Vert _{L_{p,1}({\mathbb {R}}^n)}<4^{-i}$. Denote $\tilde{f}_i=f-f_i$. Then by Corollary 3.8

$$\begin{aligned} {\mathcal {H}}^{n-(k-l)p}_\infty \left( \left\{ x\in {\mathbb {R}}^n: {\mathcal {M}}\left( |\nabla ^l \tilde{f}_i|^p\right) (x)\ge 2^{-i}\right\} \right) <C\,2^{-i}. \end{aligned}$$

Then one could repeat almost word by word the proof of [12, Theorem 3.1]. Since there are no essential differences, we omit the detailed calculations here. $\square$

### On Differentiability Properties of Sobolev–Lorentz Mappings

We start with the following simple technical observation.

#### Lemma 3.10

(see, e.g., [24, Lemma 4.1]) If $l,k\in \{1,\dots ,n\}$, $l<k$, and $v\in \mathrm {W}^{k}_{{p_\circ },1}({\mathbb {R}}^n,{\mathbb {R}}^d)$, then for any ${\varepsilon }>0$ there exists an open set $U\subset {\mathbb {R}}^n$ such that ${\mathcal {H}}^{l{p_\circ }}_\infty (U)<{\varepsilon }$ and the *uniform * convergence

$$\begin{aligned} r^{-l}\Vert 1_{B(x,r)}\cdot \nabla ^{k} v \Vert _{\mathrm {L}_{{p_\circ },1}}\rightarrow 0\qquad \text{ as } r\searrow 0 \end{aligned}$$

holds for $$x\in {\mathbb {R}}^n\setminus U$$.

#### Proof

The proof of the Lemma follows standard arguments, we reproduce it here for reader’s convenience. Fix $\sigma >0$. Let $\{ B_\alpha \}$ be a family of disjoint balls $B_{\alpha }=B(x_\alpha ,r_\alpha )$ such that

$$\begin{aligned} \Vert 1_{B_\alpha }\cdot \nabla ^{k} v\Vert _{\mathrm {L}_{{p_\circ },1}}\ge \sigma r^l_\alpha \end{aligned}$$

and $\sup _\alpha r_\alpha <\delta$ for some $\delta >0$, where $\delta$ is chosen small enough to guarantee that $\sup _\alpha \Vert 1_{B_\alpha }\cdot \nabla ^{k} v\Vert _{\mathrm {L}_{{p_\circ },1}}<1$. Then by Lemma 2.1 we have

3.19 $$\begin{aligned} \sum _\alpha r_\alpha ^{l{p_\circ }} \le \sigma ^{- {p_\circ }}\sum _\alpha \Vert 1_{B_\alpha }\cdot \nabla ^{k} v\Vert ^{{p_\circ }}_{\mathrm {L}_{{p_\circ },1}} \le \sigma ^{-{p_\circ }}\Vert 1_{\bigcup _\alpha B_\alpha }\cdot \nabla ^{k} v\Vert ^{p_\circ }_{\mathrm {L}_{{p_\circ },1}}. \end{aligned}$$

Since the last term tends to 0 as ${\mathscr {L}}^n(\bigcup _\alpha B_\alpha )\rightarrow 0$, and ${\mathscr {L}}^n(\bigcup _\alpha B_\alpha )\le c\, \delta ^{n-l{{p_\circ }}}\sum _\alpha r_\alpha ^{l{p_\circ }}$, we get easily that $\sum _\alpha r_\alpha ^{l{p_\circ }}\rightarrow 0$ as $\delta \searrow 0$. Using this fact and some standard covering lemmas, we arrive in a routine manner that for a set

$$\begin{aligned} A_{\sigma ,\delta }:=\left\{ x\in {\mathbb {R}}^n:\exists r\in (0,\delta ]\qquad r^{-l} \Vert 1_{B(x,r)}\cdot \nabla ^{k} v \Vert _{\mathrm {L}_{{p_\circ },1}} > \sigma \right\} \end{aligned}$$

the convergence

$$\begin{aligned} {\mathcal {H}}^{l{p_\circ }}_\infty \bigl (A_{\sigma ,\delta }\bigr )\rightarrow 0\qquad \text{ as } \delta \searrow 0 \end{aligned}$$

holds for any fixed $\sigma >0$. The rest part of the proof of the lemma is obvious, so we omit it. $\square$

From the last lemma (for $l=1$), Theorem 3.9 (ii) and estimate (2.7) we obtain the following result:

#### Theorem 3.11

Let $k\in \{1,\dots ,n\}$ and $v\in \mathrm {W}^{k}_{{p_\circ },1}({\mathbb {R}}^n,{\mathbb {R}}^d)$. Then there exists a Borel set $A_v\subset {\mathbb {R}}^n$ such that ${\mathcal {H}}^{p_\circ }(A_v)=0$ and for any $x\in {\mathbb {R}}^n\setminus A_v$ the function *v* is differentiable (in the classical Fréchet sense) at *x*, furthermore, the classical derivative coincides with $\nabla v(x)$ (*x* is a Lebesgue point for $\nabla v$ ).

The case $k=1$, ${p_\circ }=n$ is a classical result due to Stein [34] (see also [22]), and for $k=n$, ${p_\circ }=1$ the result is also proved in [14].

There holds the following extension of Theorem 3.11.

#### Theorem 3.12

Let $k,l\in \{1,\dots ,n\}$, $l\le k$, and $v\in \mathrm {W}^{k}_{{p_\circ },1}({\mathbb {R}}^n,{\mathbb {R}}^d)$. Then there exists a Borel set $A_v\subset {\mathbb {R}}^n$ such that ${\mathcal {H}}^{l{p_\circ }}(A_v)=0$ and for any $x\in {\mathbb {R}}^n\setminus A_v$ the function *v* is *l*-times differentiable (in the classical Fréchet–Peano sense) at *x*, i.e.,

$$\begin{aligned} \lim \limits _{r\searrow 0} \sup \limits _{y\in B(x,r)\setminus \{x\}}\frac{\bigl |v(y)-T_{v,l,x}(y)\bigr |}{|x-y|^l}=0, \end{aligned}$$

where $T_{v,l,x}(y)$ is the Taylor polynomial of order *l* for *v* centered at *x* (which is well defined ${\mathcal {H}}^{l{p_\circ }}$-a.e. by Theorem 3.9).

#### Proof

We consider only the case $l<n$; for $l=n$, the arguments are similar and becomes even simpler. Below we follow methods of [11, proof of Lemma 5.5] and [12, proof of Theorem 3.1]. By Theorem 3.9 of the present paper, there exists a set $A_l$ such that ${\mathcal {H}}^{l{p_\circ }}(A_l)=0$ and the derivatives $\nabla ^jv(x)$ are well defined for all $x\in {\mathbb {R}}^n\setminus A_l$ and $j=0,1,\dots ,l$. Further, by Lemma 3.10, there exists a sequence of open sets $U_i\subset {\mathbb {R}}^n$ such that $U_i\supset U_{i+1}$, ${\mathcal {H}}^{l{p_\circ }}_\infty (U_i)<2^{-i}$ and the *uniform * convergence

$$\begin{aligned} r^{-l}\Vert 1_{B(x,r)}\cdot \nabla ^{k} v \Vert _{\mathrm {L}_{{p_\circ },1}}\rightarrow 0\qquad \text{ as } r\searrow 0 \end{aligned}$$

holds for $x\in {\mathbb {R}}^n\setminus U_i$. It means that there exists a function $\omega _i :(0,+\infty )\rightarrow (0,+\infty )$ such that $\omega _i(r)\rightarrow 0$ as $r\searrow 0$ and

3.20 $$\begin{aligned} r^{-l}\Vert 1_{B(x,r)}\cdot \nabla ^{k} v \Vert _{\mathrm {L}_{{p_\circ },1}}\le \omega _i(r)\qquad \ \forall x\in {\mathbb {R}}^n\setminus U_i. \end{aligned}$$

Take a sequence of mappings $v_i :{\mathbb {R}}^n\rightarrow {\mathbb {R}}^d$ from Corollary 2.7, i.e., $v_i\in \mathrm {C}_0^\infty ({\mathbb {R}}^n)$ and $\Vert \nabla ^k(v-v_i)\Vert _{L_{{p_\circ },1}({\mathbb {R}}^n)}<4^{-i}$. Denote ${\tilde{v}}_i=v-v_i$ and

$$\begin{aligned} B_i=\bigl \{x\in {\mathbb {R}}^n:{\mathcal {M}}\bigl (|\nabla ^l {\tilde{v}}_i|^{p_\circ }\bigr )(x)\ge 2^{-i{p_\circ }}\bigr \},\qquad G_i=A_l\cup U_i\cup \biggl (\bigcup \limits _{j=i}^\infty B_j\biggr ). \end{aligned}$$

Then by estimate (3.18) we have

3.21 $$\begin{aligned} {\mathcal {H}}^{l{p_\circ }}_\infty (B_i)\le c 2^{-i}, \end{aligned}$$

therefore,

3.22 $$\begin{aligned} {\mathcal {H}}^{l{p_\circ }}_\infty (G_i)\le C 2^{-i}. \end{aligned}$$

By construction,

3.23 $$\begin{aligned} |\nabla ^l{\tilde{v}}_j(x)|^{p_\circ }\le {\mathcal {M}}\bigl (|\nabla ^l {\tilde{v}}_j|^{p_\circ }\bigr )(x)\le 2^{-j{p_\circ }} \end{aligned}$$

for all $$x \in {\mathbb {R}}^n \setminus G_i$$ and all $$j \ge i$$. Moreover, since $$v_j\in \mathrm {C}^\infty _0({\mathbb {R}}^n)$$, there exists constants $$M_j$$ such that $$|\nabla ^kv_j(x)|\le M_j$$$$\forall x\in {\mathbb {R}}^n$$, this fact and (3.20) implies

3.24 $$\begin{aligned} r^{-l}\Vert 1_{B(x,r)}\cdot \nabla ^{k} {\tilde{v}}_j \Vert _{\mathrm {L}_{{p_\circ },1}}\le \omega _i(r)+M_jr^{n-l}\qquad \ \forall x\in {\mathbb {R}}^n\setminus G_i. \end{aligned}$$

We start by estimating the remainder term ${\tilde{v}}_j(y)-T_{{\tilde{v}}_j,l,x}(y)$. Fix $y\in {\mathbb {R}}^n$, $x\in {\mathbb {R}}^n\setminus G_i$, $j\ge i$, and an *n*-dimensional cubic interval *Q* such that $x,y\in Q$, $|x-y|\sim \ell (Q)$. By construction and Lemma 2.10, for any multi-index $\alpha$ with $|\alpha |\le l$ we have

3.25 $$\begin{aligned} \bigl |\partial ^{\alpha }{\tilde{v}}_j(x)- \partial ^{\alpha } P_{Q,l-1}[{\tilde{v}}_j](x)\bigr |\le C\ell (Q)^{l-|\alpha |}({\mathcal {M}}\nabla ^l {\tilde{v}}_j)(x)\overset{{(3.23)}}{\le }Cr^{l-|\alpha |}2^{-j}, \end{aligned}$$

where $r=|x-y|$. Consequently,3.26Finally from the last estimate and equality $v={\tilde{v}}_j+v_j$, we have

$$\begin{aligned} | v(y)-T_{l,v,x}(y)|\le & {} |{\tilde{v}}_j(y)-T_{l,{\tilde{v}}_j,x}(y)| + |v_j(y)-T_{l,v_j,x}(y)| \\\le & {} \bigl (C_12^{-j}+\omega _i(r)+M_jr^{n-l}\bigr )r^{l}+\omega _{v_j}(r)r^{l}\\= & {} \bigl (C_12^{-j}+\omega _i(r)+M_jr^{n-l}+\omega _{v_j}(r)\bigr )r^{l}, \end{aligned}$$

where $\omega _{i}(r)\rightarrow 0$ and $\omega _{v_j}(r)\rightarrow 0$ as $r\rightarrow 0$ (the latter holds since $v_j\in \mathrm {C}^\infty _0({\mathbb {R}}^n)$ ). We emphasize that the last inequality is valid for all $y\in {\mathbb {R}}^n$, $j\ge i$, and $x\in {\mathbb {R}}^n\setminus G_i$. Therefore

$$\begin{aligned} \frac{| v(y)-T_{l,v,x}(y)|}{|x-y|^l}\rightarrow 0\qquad \text{ as } y\rightarrow x \end{aligned}$$

uniformly for all $x\in {\mathbb {R}}^n\setminus G_i$. This means, that *v* is uniformly *l*-times differentiable (in the classical Fréchet–Peano sense) at every $x\in {\mathbb {R}}^n\setminus G_i$. Then the estimate (3.22) finishes the proof. $\square$

### The Proof of the *N*-Property

In this subsection, we need to prove the assertion of Theorem 1.1 (Luzin *N*-property) for $\mathrm {W}^{k}_{{p_\circ },1}$-mappings with respect to Hausdorff content ${\mathcal {H}}^{p_\circ }_\infty$ (i.e., when $q={p_\circ }=\frac{n}{k}$ ), namely

#### Theorem 3.13

Let $k\in \{1,\dots ,n\}$, and $v\in \mathrm {W}^{k}_{{p_\circ },1}({\mathbb {R}}^n,{\mathbb {R}}^d)$. Then for each $\varepsilon >0$ there exists $\delta >0$ such that for any set $E\subset {\mathbb {R}}^n$ if ${\mathcal {H}}^{{p_\circ }}_\infty (E)<\delta$, then ${\mathcal {H}}_\infty ^{{p_\circ }}(v(E))<\varepsilon$. In particular, ${\mathcal {H}}^{{p_\circ }}(v(E))=0$ whenever ${\mathcal {H}}^{{p_\circ }}(E)=0$.

Recall that for the case $k=1$ this assertion was proved in [22], and for $k=n$ it was proved in [12], so we omit these cases. Our proof here for the remaining cases follows and expands on the ideas from [12].

For the remainder of this section, we fix $k\in \{2,\dots ,n-1\}$, and a mapping *v* in $\mathrm {W}^{k}_{{p_\circ },1}({\mathbb {R}}^n,{\mathbb {R}}^d)$. To prove Theorem 3.13, we need some preliminary lemmas that we turn to next.

Applying Corollary 3.8 for the case $p={p_\circ }=\frac{n}{k}$, $l=1$, we obtain

3.27 $$\begin{aligned} \Vert \nabla v\Vert ^{p_\circ }_{L_{p_\circ }(\mu )} \le C| | |\mu | | |_{p_\circ }\Vert \nabla ^k v\Vert ^{p_\circ }_{\mathrm {L}_{{p_\circ },1}}\quad \forall \mu \in {\mathscr {M}}^{p_\circ }, \end{aligned}$$

where *C* depends on $n,{p_\circ },d$ only.

By *a dyadic interval*, we understand a cubic interval of the form $[\frac{k_1}{2^m},\frac{k_1+1}{2^m}]\times \dots \times [\frac{k_n}{2^m},\frac{k_n+1}{2^m}]$, where $k_i,m$ are integers. The following assertion is straightforward, and hence we omit its proof here.

#### Lemma 3.14

For any *n*-dimensional cubic interval $J\subset {\mathbb {R}}^n$, there exist dyadic intervals $Q_1,\dots ,Q_{2^n}$ such that $J\subset Q_1\cup \dots \cup Q_{2^n}$ and $\ell (Q_1)=\dots =\ell (Q_{2^n})\le 2\ell (J)$.

Let $\{ Q_\alpha \}_{\alpha \in A}$ be a family of *n*-dimensional dyadic intervals. We say that the family $\{ Q_\alpha \}$ is *regular*, if for any *n*-dimensional dyadic interval *Q* the estimate

3.28 $$\begin{aligned} \ell (Q)^{{p_\circ }}\ge \sum \limits _{\alpha : Q_\alpha \subset Q}\ell (Q_\alpha )^{{{p_\circ }}} \end{aligned}$$

holds. Since dyadic intervals are either nonoverlapping or contained in one another, (3.28) implies that any regular family $\{ Q_\alpha \}$ must in particular consist of nonoverlapping intervals.

#### Lemma 3.15

(see [12, Lemma 2.3]) Let $\{ Q_\alpha \}$ be a family of *n*-dimensional dyadic intervals. Then there exists a regular family $\{ J_\beta \}$ of *n*-dimensional dyadic intervals such that $\bigcup _\alpha Q_\alpha \subset \bigcup _\beta J_\beta$ and

$$\begin{aligned} \sum \limits _{\beta }\ell (J_\beta )^{{{p_\circ }}}\le \sum \limits _{\alpha }\ell ( Q_\alpha )^{{{p_\circ }}}. \end{aligned}$$

#### Lemma 3.16

For each $\varepsilon >0$ there exists $\delta =\delta (\varepsilon ,v)>0$ such that for any regular family $\{ Q_\alpha \}$ of *n*-dimensional dyadic intervals we have if

3.29 $$\begin{aligned} \sum _\alpha \ell (Q_\alpha )^{{p_\circ }}<\delta , \end{aligned}$$

then

3.30 $$\begin{aligned} \sum _\alpha \Vert 1_{Q_\alpha }\cdot \nabla ^{k} v\Vert ^{{{p_\circ }}}_{\mathrm {L}_{{p_\circ },1}}<\varepsilon \end{aligned}$$

and

3.31 $$\begin{aligned} \sum _\alpha \frac{1}{\ell (Q_\alpha )^{n-{p_\circ }}}\int _{Q_\alpha }|\nabla v|^{{{p_\circ }}}<\varepsilon . \end{aligned}$$

#### Proof

Fix $\varepsilon \in (0,1)$ and let $\{ Q_\alpha \}$ be a regular family of *n*-dimensional dyadic intervals satisfying (3.29), where $\delta >0$ will be specified below.

Let us start by checking (3.30). We have

$$\begin{aligned} \sum _\alpha \Vert 1_{Q_\alpha }\cdot \nabla ^{k} v\Vert ^{{{p_\circ }}}_{\mathrm {L}_{{p_\circ },1}}\overset{{\small {\mathrm{Lemma}}~2.1}}{\le }\Vert 1_{\bigcup _\alpha Q_\alpha }\cdot \nabla ^{k} v\Vert ^{p_\circ }_{\mathrm {L}_{{p_\circ },1}}. \end{aligned}$$

Using (2.2), we can rewrite the last estimate as

3.32 $$\begin{aligned} \sum _\alpha \Vert 1_{Q_\alpha }\cdot \nabla ^{k} v\Vert ^{{{p_\circ }}}_{\mathrm {L}_{{p_\circ },1}}\le \biggl ( \int _0^{+\infty }\bigl [{\mathscr {L}}^n(\{x\in {\bigcup _\alpha Q_\alpha }: \, |\nabla ^{k} v(x)|>t\})\bigr ]^{\frac{1}{{p_\circ }}} \, \mathrm{d}t\biggr )^{p_\circ }. \end{aligned}$$

Since

$$\begin{aligned} \int _0^{+\infty }\bigl [{\mathscr {L}}^n(\{x\in {\mathbb {R}}^n:|\nabla ^{k} v(x)|>t\})\bigr ]^{\frac{1}{{p_\circ }}} \, \mathrm{d}t <\infty , \end{aligned}$$

it follows that the integral on the right-hand side of (3.32) tends to zero as ${\mathscr {L}}^n(\bigcup _\alpha Q_\alpha )$ tends to zero. In particular, it will be less than ${\varepsilon }$ if the condition (3.29) is fulfilled with a sufficiently small $\delta$. Thus (3.30) is established for all $\delta \in (0,\delta _{1}]$, where $\delta _{1}=\delta _{1}(\varepsilon ,v)>0$.

Next we check (3.31). By virtue of Corollary 2.7, applied coordinate-wise, we can find a decomposition $v=v_0+v_1$, where $\Vert \nabla v_{0} \Vert _{\mathrm {L}^{\infty }} \le K = K(\varepsilon ,v)$ and

3.33 $$\begin{aligned} \Vert \nabla ^{k} v_{1} \Vert _{\mathrm {L}_{{p_\circ },1}} < \varepsilon . \end{aligned}$$

Assume that $\delta \in (0,\delta _{1}]$ and

3.34 $$\begin{aligned} \sum _\alpha \ell (Q_\alpha )^{{{p_\circ }}}< \delta < \tfrac{1}{K^{{{p_\circ }}}+1}\varepsilon . \end{aligned}$$

Define the measure $\mu$ by

3.35 $$\begin{aligned} \mu = \left( \sum _\alpha \frac{1}{\ell (Q_\alpha )^{n-{{p_\circ }}}} 1_{Q_\alpha }\right) {{\mathscr {L}}}^{n}, \end{aligned}$$

where $1_{Q_\alpha }$ denotes the indicator function of the set $Q_\alpha$.

**Claim** The estimate

3.36 $$\begin{aligned} \sup _{J}\bigl \{ \ell (J)^{-{{p_\circ }}}\mu (J)\bigr \} \le 2^{n+{{p_\circ }}} \end{aligned}$$

holds, where the supremum is taken over all *n*-dimensional cubic intervals.

Indeed, write for a dyadic interval *Q*

$$\begin{aligned} \mu (Q) =\sum _{\alpha : Q_\alpha \subset Q} \ell (Q_\alpha )^{{{p_\circ }}}+\sum _{\alpha : Q_\alpha \nsubseteq Q}\ \frac{\ell (Q \cap Q_{\alpha })^n}{\ell (Q_\alpha )^{n-{{p_\circ }}}}. \end{aligned}$$

By regularity of $\{ Q_{\alpha } \}$ the first sum is bounded above by $\ell (Q)^{{p_\circ }}$. If the second sum is nonzero, then there must exist an index $\alpha$ such that $Q_{\alpha } \nsubseteq Q$ and $Q_\alpha$, *Q* overlap. But as the intervals $\{ Q_\alpha \}$ are nonoverlapping and dyadic, we must then precisely have one such interval $Q_\alpha$ and $Q_\alpha \supset Q$. But then the first sum is empty and the second sum has only the one term $\ell (Q)^{n}/\ell (Q_{\alpha })^{n-{p_\circ }}$, hence is at most $\ell (Q)^{{p_\circ }}$. Thus the estimate $\mu (Q)\le \ell (Q)^{{p_\circ }}$ holds for every dyadic *Q*. The inequality (3.36) in the case of a general cubic interval *J* follows from the above dyadic case and Lemma 3.14. The proof of the claim is complete.

Now return to (3.31). By properties (3.27), (3.33),, and (3.34) (applied to the mapping $v_1$ ), we have

$$\begin{aligned} \sum _\alpha \frac{1}{\ell (Q_\alpha )^{n-{p_\circ }}}\int _{Q_\alpha }|\nabla v|^{{{p_\circ }}}\le & {} \frac{2^{{{p_\circ }}-1}K^{{{p_\circ }}}}{K^{{{p_\circ }}}+1}{\varepsilon }+ \sum _{\alpha } \frac{2^{{{p_\circ }}-1}}{\ell (Q_\alpha )^{n-{{p_\circ }}}}\int _{Q_\alpha } |\nabla v_1|^{{{p_\circ }}}\\\le & {} C' \left( \varepsilon +\int |\nabla v_1|^{{{p_\circ }}} \,d\mu \right) \le C''\varepsilon . \end{aligned}$$

Since $\varepsilon >0$ was arbitrary, the proof of Lemma 3.16 is complete. $\square$

#### Proof of Theorem 3.13

Fix $\varepsilon >0$ and take $\delta =\delta (\varepsilon ,v)$ from Lemma 3.16. Then by Corollary 2.3 for any regular family $\{ Q_\alpha \}$ of *n*-dimensional dyadic intervals we have if $\sum _\alpha \ell (Q_\alpha )^{{{p_\circ }}}<\delta$, then $\sum _\alpha \bigl (\mathop {\mathrm{diam}}v(Q_\alpha )\bigr )^{{{p_\circ }}}<C\varepsilon$. Now we may conclude the proof of Theorem 3.13 by use of Lemmas 3.14 and 3.15. Indeed they allow us to find a $\delta _0 >0$ such that if for a subset *E* of ${\mathbb {R}}^n$ we have ${\mathcal {H}}^{{{p_\circ }}}_\infty (E)<\delta _0$, then *E* can be covered by a regular family $\{ Q_\alpha \}$ of *n*-dimensional dyadic intervals with $\sum _\alpha \ell (Q_\alpha )^{{{p_\circ }}}<\delta$. $\square$

#### Remark 3.17

Note that the order of integrability ${p_\circ }$ is sharp: for example, the Luzin *N*-property fails in general for continuous mappings $v\in \mathrm {W}^1_n({\mathbb {R}}^n,{\mathbb {R}}^n)$ (here $k=1,q={p_\circ }=n$), see, e.g., [26].

### Morse–Sard–Dubovitskiĭ–Federer Theorem for Sobolev Mappings

Let $k,m\in \{1,\dots ,n\}$ and $v\in \mathrm {W}^{k}_{{p_\circ },1, {\mathrm{loc}}}(\Omega ,{\mathbb {R}}^d)$, where $\Omega$ is an open subset of ${\mathbb {R}}^n$. Then, by Theorem 3.9 (ii), there exists a Borel set $A_v$ such that ${\mathcal {H}}^{p_\circ }(A_v)=0$ and all points of the complement $\Omega \setminus A_v$ are $\mathrm {L}_{{p_\circ }}$-Lebesgue points for the gradient $\nabla v(x)$. Moreover, *v* is differentiable (in the classical Fréchet sense) at every point of $\Omega \setminus A_v$.

Denote $Z_{v,m}=\{x\in \Omega \setminus A_v:\mathop {\mathrm{rank}}\nabla v(x)<m\}$. The purpose of this section is to prove the assertion of Theorem 1.5:

3.37 $$\begin{aligned} {\mathcal {H}}^{q_\circ }(v(Z_{v,m}))=0. \end{aligned}$$

The exponents occurring in the theorem are the critical exponents that were defined in (1.6):

$$\begin{aligned} {p_\circ }= \frac{n}{k} \quad \text{ and } \quad {q_\circ }= m-1+\frac{n- m+1}{k}. \end{aligned}$$

By an easy calculation, assumptions ${n\ge } m\ge 1$, $k\ge 1$ imply

3.38 $$\begin{aligned} {p_\circ }\le {q_\circ }\le n. \end{aligned}$$

Note that in the double inequality (3.38), we have equality in the first inequality iff $m=1$ or $k=1$, while in the second inequality equality holds if and only if $k=1$. In particular,

$$\begin{aligned} {p_\circ }< {q_\circ }< n\quad \text{ for } k,m\in \{2,\dots ,n\}. \end{aligned}$$

By results obtained in the previous papers [11, 12, 24] (see commentary to the Theorem 1.5 in the Introduction), we need only consider the case

$$\begin{aligned} m=1,\quad {q_\circ }={p_\circ }=\frac{n}{k}. \end{aligned}$$

Before embarking on the detailed proof, let us make some preliminary observations that will enable us to make some convenient additional assumptions. Namely because the result is local we can without loss in generality assume that $\Omega = {\mathbb {R}}^n$. For the remainder of the section we fix $k \in \{ 2, \dots \, n \}$ and a mapping $v \in \mathrm {W}^{k}_{{p_\circ },1}({\mathbb {R}}^n , {\mathbb {R}}^d )$. In view of the definition of critical set we have for $m=1$

$$\begin{aligned} Z_v=Z_{v,1} = \left\{ x\in {\mathbb {R}}^n\setminus A_v:\nabla v(x)=0\right\} . \end{aligned}$$

The following lemma provides the main step in the proof of Theorem 1.5.

#### Lemma 3.18

For any *n*-dimensional dyadic interval $Q\subset {\mathbb {R}}^n$ the estimate

3.39 $$\begin{aligned} {\mathcal {H}}^{{p_\circ }}_\infty (v(Z_v\cap Q))\le C\,\Vert \nabla ^{k} v\Vert ^{{p_\circ }}_{\mathrm {L}_{{p_\circ },1}(Q)} \end{aligned}$$

holds, where the constant *C* depends on *n*, *m*, *k*, *d* only.

#### Proof

By virtue of (2.5) it suffices to prove that

3.40 $$\begin{aligned} {\mathcal {H}}^{{p_\circ }}_\infty (v(Z_v\cap Q))\le C\Vert \nabla ^{k} v_Q\Vert ^{{p_\circ }}_{\mathrm {L}_{{p_\circ },1}({\mathbb {R}}^n)} \end{aligned}$$

for the mapping $v_{Q}$ defined in Lemma 2.2, where $C=C(n,m,k,d)$ is a constant. To establish (3.40), it is possible to repeat almost verbatim the proof of Lemma 3.2 in [24]. One must observe the following minor changes: first ${q_\circ }={p_\circ }$, and next, instead of Corollary 1.8 from [24] one must use Corollary 3.8 established above. Note that in the present situation the calculations simplify since for $m=1$ many of terms from [24, proof of Lemma 3.2] disappear. $\square$

#### Corollary 3.19

For any $\varepsilon >0$ there exists $\delta >0$ such that for every subset *E* of ${\mathbb {R}}^n$ we have ${\mathcal {H}}^{{p_\circ }}_\infty (v(Z_v\cap E))\le \varepsilon$ provided ${\mathscr {L}}^n(E)\le \delta$. In particular, ${\mathcal {H}}^{{p_\circ }}(v(Z_v\cap E))=0$ whenever ${\mathscr {L}}^n(E)=0$.

#### Proof

Let ${\mathscr {L}}^n(E)\le \delta$, then we can find a family of nonoverlapping *n*-dimensional dyadic intervals $Q_\alpha$ such that $E\subset \bigcup _\alpha Q_\alpha$ and $\sum \limits _\alpha \ell ^n(Q_\alpha )<C\delta$. Then in view of Lemma 2.1 we have

3.41 $$\begin{aligned} \sum _\alpha \Vert \nabla ^{k} v\Vert ^{{p_\circ }}_{\mathrm {L}_{{p_\circ },1}(Q_\alpha )}\le \Vert \nabla ^{k} v\Vert ^{p_\circ }_{\mathrm {L}_{{p_\circ },1}(\bigcup Q_\alpha )} \end{aligned}$$

This estimate together with Lemma 3.18 allow us to conclude the required smallness of

$$\begin{aligned} \sum _\alpha {\mathcal {H}}^{{p_\circ }}_\infty (Z_v\cap Q_\alpha )\ge {\mathcal {H}}^{{p_\circ }}_\infty (Z_v\cap E). \end{aligned}$$

$$\square $$

Invoking Dubovitskiĭ–Federer’s Theorem (see commentary to the Theorem 1.5 in the Introduction) for the smooth case $g\in \mathrm {C}^{k}({\mathbb {R}}^n,{\mathbb {R}}^d)$, Theorem 3.9 (iii) (applied to the case $l=k$ ) implies

#### Corollary 3.20

(see, e.g., [13]) There exists a set $\widetilde{Z}_{v}$ of *n*-dimensional Lebesgue measure zero such that ${\mathcal {H}}^{{p_\circ }}(v(Z_v\setminus \widetilde{Z}_{v}))=0$. In particular, ${\mathcal {H}}^{{p_\circ }}(v(Z_v))={\mathcal {H}}^{{p_\circ }}(v(\widetilde{Z}_{v}))$.

From Corollaries 3.19 and 3.20, we conclude that ${\mathcal {H}}^{{p_\circ }}(v(Z_v))=0$, and this ends the proof of Theorem 1.5.

## Footnotes

1 The above estimate (1.2) remains valid for $q=p$ if the measure $\mu$ instead of (1.1) satisfies the estimate $\mu (K)\le C R_{{\alpha },p}(K)$ for all compact sets $K\subset {\mathbb {R}}^n$, here $R_{{\alpha },p}$ is the Riesz capacity: $R_{{\alpha },p}(K)=\inf \{\Vert f\Vert _{L_p}: f\in L_p({\mathbb {R}}^n),\ I_{\alpha }f(x)\ge 1$ on *K*}, see [3]. Another geometric criterion for such an estimate (without using of Riesz capacity) was found in [23]. A simpler sufficient condition was found in [19], see also [29, p. 28].

2 Federer announced [17] his result in 1966, this announcement (without any proofs) was sent on 08.02.1966. For the historical details, Dubovitskiĭ sent his paper [15] (with complete proofs) a month earlier, on 10.01.1966.
